# Experimental data on demographic, functional and structures of patients with schizophrenia and schizophrenia-dementia

**DOI:** 10.1016/j.dib.2020.106286

**Published:** 2020-09-07

**Authors:** Juan Rivas, Jose Libreros, Maria Trujillo, Aura Hurtado, Joan Camprodon

**Affiliations:** a Psychiatry Unit, Fundación Valle del Lili-Universidad ICESI, Kra. 98 No 18-49, Cali, Colombia; b Hospital Psiquiátrico Universitario del Valle, Calle 5 No, 80 - 00, Cali, Colombia; c Department of Psychiatry, Universidad del Valle, Ciudad Universitaria San Fernando, Calle 4B No 36B-37, Cali 760043, Colombia; d School of Systems Engineering and Computing, Universidad del Valle, Ciudad Universitaria Meléndez, Calle 13 No 100-00, Cali 760032, Colombia; e Department of Psychiatry, Massachusetts General Hospital, 55 Fruit Street Boston, MA 02114, United States

**Keywords:** Schizophrenia, Schizophrenia with dementia, Neuropsychological profiles, Measurements of brain morphometry using MRI

## Abstract

Schizophrenia is a severe mental disorder that includes behavioural and cognitive manifestations generated by genetic or environmental factors, caused by a dysfunction of the dopaminergic system which contributes to the genesis of psychosis, producing a profound effect on affected individuals and society [6]. In this work, demographic data, neuropsychological profiles and measurements of brain morphometry, using Magnetic Resonance Image (MRI), of three groups of patients are presented. A control group with 15 patients, a schizophrenic without dementia group with 10 patients, and a schizophrenic with dementia group with 10 patients constituted the observed sample. Results of 21 neuropsychological tests and 11 brain structure measurements are included. The data set is a comprehensive source for advancing in a further understanding of schizophrenia and schizophrenia-dementia neuro-pathologies.

**Specifications Table**

**Table:** 

Subject	Medicine::Medicine (General)
Specific subject area	Psychiatry
Type of data	Figure and table
How data were acquired	Two general scanning tests were used, the Mini Mental State Examination (MMSE) and the Addenbrooke's Cognitive Examination (ACE). Additionally, to confirm or rule out the presence of a dementia state, the Clinical Dementia Rating (CDR) Scale and the Hachinski Ischemic Score were administered. All participants also answered the Yesavage Geriatric Depression Scale, with the aim of analysing possible concomitance. In schizophrenic patients the Positive and Negative Symptom Scale in Schizophrenia (PANSS) was applied. To analyze the mnemic component, the Hopkins Verbal Learning Test (HVLT), the King's Complex Figure test, and the Free and Cued Selective Reminding Test (FCSRT) were used. The Boston Naming Test was used to test language function. In relation to functions associated with the prefrontal cortex, the Trail Making Test - A (TMT - A), the Phonological Fluency test (Letter F and S), the Semantic Fluency test (animal fluency) and the Digit Spam test were applied. The Phonological Fluency test (Letter F and S) and the Semantic Fluency test (animal fluency) were used to assess mental flexibility and the ability to categorize. Regarding brain morphometry, Magnetic Resonance Images (MRI) were acquired using a SIEMENS AVANTO scanner at the Fundación Valle del Lili, FreeSurfer recon-all, software suite, was used to cortical reconstruction process, using T2.
Data format	Raw and order statistics
Parameters for data collection	We included subjects who met the criteria of the Diagnostic and Statistical Manual of Mental Disorders, in the fifth edition (DSM-V), had a Mini Mental (MMSE) greater than or equal to 26 and a Clinical Dementia Rating (CDR) score less than 0,5 for the schizophrenics without dementia group. The schizophrenics with dementia group met the DSM-V criteria for schizophrenia, had a MMSE less than 26 and a CDR greater than 1. The control group had no history of mental illness and the MMSE was greater than 26 and the CDR was 0. Exclusion criteria for all patients were history of other neurological diseases such as epilepsy, stroke, history of head trauma, HIV infection or neurological diseases.
Description of data collection	A sample of 35 patients 10 schizophrenics without dementia, 10 schizophrenics with dementia, and 15 without schizophrenia and dementia as a control group was selected with a non-probability sampling. All individuals were over 45 years of age, were ambulatory and were recruited at the Hospital Psiquiátrico Universitario del Valle, in Cali - Colombia (schizophrenics without dementia and with dementia groups) and at the Fundación Valle del Lili (control group). The control group was taken from a study of the progression of Mild Cognitive Impairment. The onset of symptoms was before 30 years of age in all patients. The study was approved by the Ethics Committees of the Universidad del Valle and the Fundación Valle del Lili and all individuals signed the informed consent form before being included in the study.
Data source location	Neuropsychological tests were conducted at the Hospital Psiquiátrico Universitario del Valle in Cali, Colombia; MRI scans were acquired at the Image Diagnostic Unit of the Fundación Valle del Lili in Cali, Colombia; and volume measurements of brain structures were done at the Laboratory for Computational Neuroimaging, Harvard Medical School, Boston, USA.
Data accessibility	Processing data is provided in this article and original data is available at https://zenodo.org/record/3901876#.Xu1w2G5FxPb

## Value of the Data

● Neuropsychological profiles and measurements of brain morphometry may provide an opportunity for better understanding relations between brain morphology and cognitive function.
● Magnetic Resonance Images, commonly used to visually supporting diagnostics, may be extended to a source of measurements of brain morphometry for modeling longitudinal changes in brain structure.
● Data presented in this work may be used in researches on neurological sciences, psychiatry and related areas.
● From a clinical point of view data gives important information on cognitive profile from people with schizophrenia and dementia.

## 1 Data Description

**Fig. 1:**
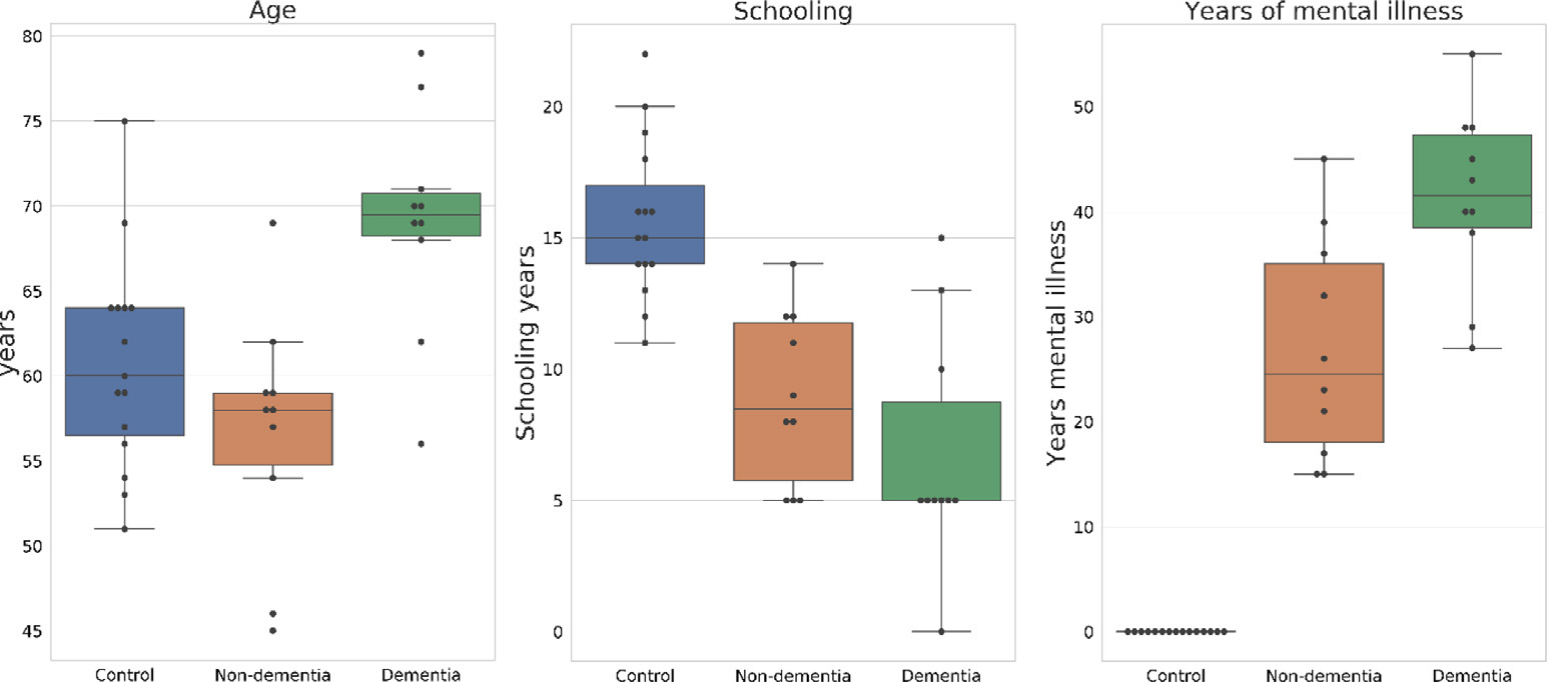
Data distribution of demographic variables.

Data presented in this work includes demographic basic information and the results of 21 neuropsychological tests and 172 measurements of brain morphometry by side (344 both sides) derived from 35 patients distributed as follow: a control group with 15 patients (9 female and 6 male), a schizophrenics without dementia group with 10 patients (5 female and 5 male) and a schizophrenics with dementia group with 10 patients (4 female and 6 male). The demographic data is presented using box-plots, in Fig. 1, to allow analysing data distribution. Original data are available at https://zenodo.org/record/3901876#.Xu1w2G5FxPb

Neuropsychological tests were applied at the Hospital Psiquiátrico Universitario del Valle. The applied neuropsychological tests are: the Mini Mental State Examination (MMSE), the Addenbrooke's Cognitive Examination (ACE), the Clinical Dementia Rating (CDR) Scale, the Hachinski Ischemic Score (HIS), the Yesavage Geriatric Depression Scale (YGD), the Positive and Negative Symptom Scale in Schizophrenia (PANSS), the Hopkins Verbal Learning Test (HVLT), the Rey Complex Figure test (RCFT), the Free and Cued Selective Reminding Test (FCSRT), the Boston Naming Test (BNT), the Trail Making Test - A (TMT - A), the Phonological Fluency test (Letter F and S), the Semantic Fluency test (animal fluency) (SFT), the Digit Spam test (DST). An inside of the purposes of these tests is in Table 1.

**Table 1:** Predominant evaluated cognitive functions.

Tests	Predominant evaluated functions
LetterF Animal Fluency LetterS	Verbal fluency, linguistic categorization, verbal memory attention
HVLT Palabras de Rey-Total Recall HVLT-Delayed Recall	Audioverbal memory - short term
Rey Figure-Copy	Visual-spatial – graphic analysis and synthesis
Rey Figure-Immediate Recall Rey Figure-Delayed Recalled	Visual memory - short term
Digit Span	Operational memory
Boston Naming Test	Semantic memory
FCSRT-IDEN	Semantic memory
FCSRT-Free Recall score FCSRT-Cued Recall score FCSRT-Total recall score	Audioverbal memory - short term

**Fig. 2:**
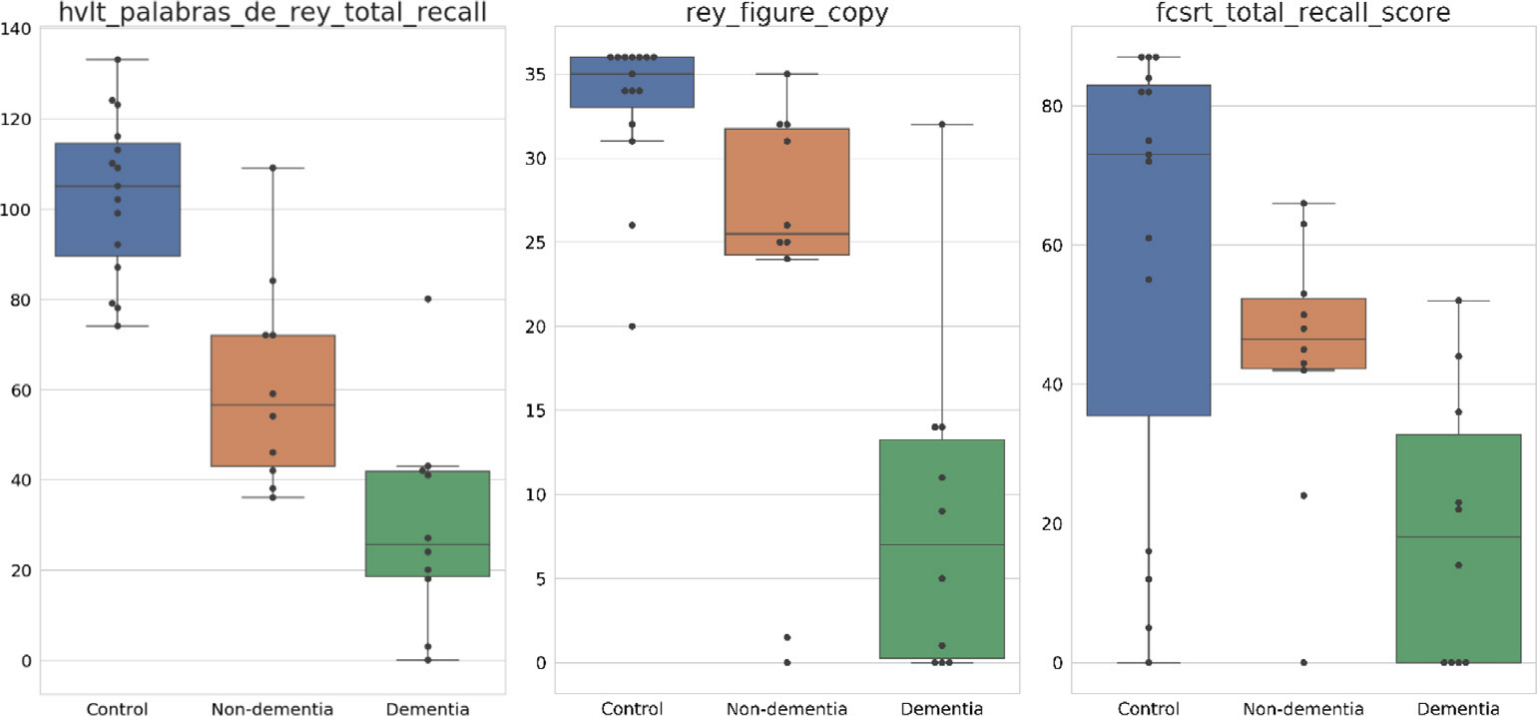
Data distribution of scores from HVLT Palabras de Rey total recall, Rey Figure-Copy and FCSRT total recall.

The scores of HVLT Palabras de Rey total recall, Rey Figure-Copy and FCSRT total recall score tests are presented using box-plots, in Fig. 2, to illustrate data distribution.

Table 2 presents order statistics – where Q1 is the first quartile, Q2 is the second quartile and Q3 is the third quartile – of the obtained scores of the neuropsychological tests per group. Original data is available at https://zenodo.org/record/3901876#.Xu1w2G5FxPb.

**Table 2:** Order statistics of the neuropsychological test scores.

TEST	Control			Schizophrenics No-Dementia			Schizophrenics With-Dementia		
	Q1	Q2	Q3	Q1	Q2	Q3	Q1	Q2	Q3
Depression	0,00	1,00	2,00	2,00	2,50	8,00	1,00	3,00	6,00
Hachinski_ischemia_score	0,00	1,00	2,75	1,00	2,00	2,00	1,00	2,00	3,00
Cdr	0,00	0,00	0,50	0,00	0,75	1,50	2,00	2,00	3,50
Ace	94,30	98,00	99,00	69,00	78,50	88,00	38,00	46,00	63,00
Mmse	30,00	30,00	30,00	25,00	26,00	27,00	13,00	17,00	22,00
Letter_f	4,30	6,00	7,00	4,00	4,00	5,00	0,00	0,00	2,00
Animal_fluency	6,25	7,00	7,00	2,00	3,00	4,00	0,00	0,00	2,00
Letter_s	4,30	6,00	7,00	0,00	3,00	4,00	0,00	0,00	0,00
Hvlt_palabras_de_rey_total_recall	88,30	105,00	115,30	42,00	56,50	72,00	18,00	25,50	42,00
Hvlt_delayed_recall	9,00	11,00	13,80	4,00	4,50	7,00	0,00	0,00	3,00
Rey_figure_copy	32,50	35,00	36,00	24,00	25,50	32,00	0,00	7,00	14,00
Rey_figure_immediate_recall	14,60	18,00	27,30	0,00	7,50	11,00	0,00	0,00	3,00
Rey_figure_delayed_recalled	14,00	18,00	22,40	0,50	7,50	13,00	0,00	0,00	1,00
Digit_span	4,00	5,00	6,75	3,00	4,00	4,00	2,00	2,50	3,00
Boston_naming_test	19,00	19,00	20,00	0,00	8,00	19,00	0,00	11,50	15,00
Tmt_a	34,50	39,00	54,50	76,00	103,50	175,00	126,00	126,00	126,00
Tmt_b	75,00	110,00	122,50	188,00	259,00	259,00	150,00	150,00	150,00
Forst_iden	16,00	16,00	16,00	14,00	15,00	16,00	10,00	12,50	15,00
Fcsrt_free_recall_score	25,00	35,00	39,80	13,00	18,00	26,00	0,00	8,50	13,00
Fcsrt_cued_recall_score	38,50	43,00	45,50	19,00	24,00	30,00	0,00	7,00	19,00
Fcsrt_total_recall_score	25,80	73,00	83,50	42,00	46,50	53,00	0,00	18,00	36,00

MRI scans were taken using a Siemens Avanto 1,5 Tesla MRI scanner, at the Diagnostic Images Unit of the Fundación Valle del Lili. FreeSurfer was used to measure brain morphometry on MRI. Tables 3 to 12 present order statistics of the brain morphometry measurements by side per group – where Q1 is the first quartile, Q2 is the second quartile and Q3 is the third quartile. Original data is available at https://zenodo.org/record/3901876#.Xu1w2G5FxPb.

**Table 3:** Measurements of brain morphometry: Segmentation.

SEGMENTATION	Control			Schizophrenics No-Dementia			Schizophrenics With-Dementia		
	Q1	Q2	Q3	Q1	Q2	Q3	Q1	Q2	Q3
Left-Accumbens-area	372,15	398,15	547,33	365,28	443,25	466,85	296,28	356,55	411,53
Right-Accumbens-area	355,48	410,90	500,98	381,90	431,45	453,25	309,20	349,70	397,25
Left-CortexVol	193,703,98	210,425,24	235,104,94	195,817,00	213,996,24	221,597,1	166,594,66	183,026,25	196,310,83
Right-CortexVol	195,884,58	210,385,64	235,334,98	193,751,25	209,693,53	223,193,58	168,818,72	185,597,16	200,318,73
Left-CerebralWhite MatterVol	210,616,22	220,292,89	236,064,99	215,440,21	238,384,12	262,657,87	196,199,91	223,277,58	247,728,70
Right-Cerebral WhiteMatterVol	207,572,81	219,276,01	238,516,99	214,819,93	240,027,19	258,727,12	197,910,77	220,865,36	244,845,96

**Table 4:** Measurements of brain morphometry: General Segmentation.

GENERAL-SEGMENTACION	Control			Schizophrenics No-Dementia			Schizophrenics With-Dementia		
	Q1	Q2	Q3	Q1	Q2	Q3	Q1	Q2	Q3
CC_Posterior	910,88	959,00	1024,85	770,25	883,40	1117,60	813,80	896,70	941,78
CC_Mid_Posterior	479,08	534,2	589,18	348,70	407,75	493,70	368,45	406,45	469,70
CC_Central	447,08	470,1	573,1	381,03	437,05	548,83	344,00	393,2	457,53
CC_Mid_Anterior	461,18	475,00	511,75	415,28	446,15	546,70	329,95	365,10	447,78
CC_Anterior	711,73	836,75	960,98	694,00	825,55	957,6	698,58	816,10	938,35
BrainSegVol	1,018,700,50	1,053,380,50	1,175,473,00	1,015,547,50	1,126,853,50	1,184,616,00	961,482,50	1,011,666,00	1,077,947,75
BrainSegVolNotVent	990,870,00	1,040,587,50	1,129,266,50	989,653,75	1,101,573,50	1,125,980,00	918,655,00	952,374,50	1,042,539,75
BrainSegVolNotVentSurf	990,822,82	1,040,537,78	1,128,862,90	988,864,77	1,101,123,95	1,126,025,36	918,344,91	952,257,67	1,042,905,24
CortexVol	389,588,56	420,810,88	470,439,92	389,568,25	423,689,77	445,127,01	335,413,37	369,071,85	393,223,51
CerebralWhiteMatterVol	418,189,03	439,568,90	475,061,25	430,260,15	479,115,01	521,384,99	394,110,68	444,142,94	492,574,66
SubCortGrayVol	48,103,25	52,360,00	55,914,75	49,122,00	52,492,00	57,321,75	44,138,25	47,385,5	50,815,00
TotalGrayVol	544,958,93	576,663,88	630,767,92	532,978,78	578,038,27	594,371,26	472,235,62	506,911,58	537,076,37
SupraTentorialVol	889,743,35	924,298,78	1,038,298,90	908,140,02	983,006,95	1,068,239,61	853,214,41	899,385,09	958,100,99
SupraTentorialVolNotVent	865,523,82	914,636,28	996,551,90	886,437,77	961,568,95	1,016,016,86	815,163,66	846,491,4	927,314,74
BrainSegVol-to-eTIV	0,71	0,75	0,77	0,68	0,70	0,75	0,67	0,70	0,74
EstimatedTotalIntraCranialVol	1,351,869,10	1,436,059,90	1,541,447,93	1,409,528,18	1,545,802,93	1,730,606,82	1,339,112,84	1,464,662,54	1,558,891,35

**Table 5:** Measurements of brain morphometry: Basal Ganglia.

BASAL GANGLIA	Control			Schizophrenics No-Dementia			Schizophrenics With-Dementia		
	Q1	Q2	Q3	Q1	Q2	Q3	Q1	Q2	Q3
Left- thalamusproper	6486,10	7117,10	7771,5	6202,40	6805,50	7242,20	5415,20	5613,40	5913,00
Right- thalamusproper	6241,90	6490,75	7218,10	6045,60	6754,40	6941,10	5273,10	5817,40	6119,10
Left-caudate	2854,00	3131,35	3391,00	2799,30	3334,00	3625,80	2979,50	3242,15	3678,70
Right-caudate	2772,60	3134,85	3405,80	2941,70	3191,50	3541,50	2968,00	3307,75	3455,90
Left-putamen	3908,60	4163,45	4558,90	4170,00	4302,40	4997,80	3475,00	3883,90	4327,30
Right-putamen	3755,60	4065,20	4608,80	3963,50	4317,30	4910,10	3280,70	3976,40	4116,80
Left- pallidum	1480,90	1608,95	1925,20	1898,50	1957,35	2073,80	1380,70	1753,00	1846,20
Right- pallidum	1578,40	1658,65	1825,40	1890,10	2000,15	2116,60	1460,90	1788,60	1881,70

**Table 6:** Measurements of brain morphometry: Ventricles.

VENTRICLES	Control			Schizophrenics No-Dementia			Schizophrenics With-Dementia		
	Q1	Q2	Q3	Q1	Q2	Q3	Q1	Q2	Q3
Left-lateralVentricle	5629,40	7028,15	9702,80	9099,70	10,075,35	21,686,80	10,714,80	17,667,70	21,460,30
Right-lateralVentricle	5042,60	6398,40	9421,60	9156,30	10,627,00	25,731,00	10,382,90	15,050,45	21,391,20
Inf-Lat-Vent	230,20	335,75	446,60	269,30	637,40	776,30	596,70	980,70	1555,40
3rd-Ventricle	874,00	978,00	1142,30	1181,10	1576,80	2553,70	1753,20	2091,75	2671,10
4th-Ventricle	1410,60	1566,85	1922,00	1381,40	1746,55	2178,60	1558,30	1645,15	1981,70
CSF	740,70	827,30	971,8	911,70	1188,30	1492,30	983,10	1147,15	1368,10

**Table 7:** Measurements of brain morphometry: Hippocampus.


HIPPOCAMPUS	Control			Schizophrenics No-Dementia			Schizophrenics With-Dementia		
	Q1	Q2	Q3	Q1	Q2	Q3	Q1	Q2	Q3
Left-Hippocampal_tail	520,98	533,18	589,86	461,68	502,17	567,30	448,90	491,29	499,77
Left-subiculum-body	217,02	221,75	235,90	195,03	209,46	260,00	194,18	198,47	226,45
Left-CA1-body	106,86	118,81	138,85	89,04	108,48	121,41	98,56	102,63	106,44
Left-subiculum-head	159,40	184,99	203,19	165,00	178,94	223,86	135,71	165,25	178,45
Left-hippocampal-fissure	112,16	138,38	161,35	130,81	137,30	163,70	145,99	155,39	174,02
Left-presubiculum-head	122,46	134,10	146,92	114,47	120,82	149,31	100,31	109,17	118,95
Left-CA1-head	444,45	474,02	519,82	403,13	460,11	490,81	347,13	384,23	468,92
Left-presubiculum-body	146,12	153,46	171,83	128,46	147,79	162,73	110,48	125,06	167,60
Left-parasubiculum	55,11	56,16	62,63	43,44	50,60	66,06	37,46	51,01	65,79
Left-molecular_layer_HP-head	294,89	301,21	332,05	258,57	305,47	326,21	235,94	247,79	271,19
Left-molecular_layer_HP-body	201,47	213,21	226,23	173,82	192,83	226,90	159,34	181,25	195,97
Left-GC-ML-DG-head	130,23	138,81	151,34	120,66	131,24	142,69	93,19	111,15	114,01
Left-CA3-body	72,467	83,18	103,25	65,29	75,73	91,63	62,29	77,46	87,89
Left-GC-ML-DG-body	125,11	129,49	136,34	106,84	116,42	135,18	95,44	113,38	116,62
Left-CA4-head	107,12	116,68	124,51	100,79	110,37	124,89	77,96	92,76	97,19
Left-CA4-body	110,93	116,19	123,70	94,81	106,29	123,20	88,88	103,30	105,26
Left-fimbria	59,70	66,43	73,78	71,62	81,87	86,21	37,66	50,07	58,16
Left-CA3-head	97,24	109,58	122,43	92,15	99,03	126,25	70,78	75,075	92,44
Left-HATA	46,22	52,55	59,00	39,43	53,39	57,32	37,34	41,60	45,90
Left-Whole_hippocampal_body	1067,09	1122,19	1176,67	970,99	1083,85	1149,84	859,85	966,51	1031,34
Left-Whole_hippocampal_head	1478,46	1553,40	1687,63	1310,81	1545,42	1666,40	1187,85	1272,62	1432,99
Left-Whole_hippocampus	3080,16	3250,59	3393,37	2742,31	3191,60	3224,74	2477,23	2744,36	2896,62
Right-Hippocampal_tail	539,65	580,54	621,54	460,70	505,65	581,44	451,86	510,067	538,25
Right-subiculum-body	219,54	231,92	257,21	211,12	227,11	240,61	179,08	199,97	208,28
Right-CA1-body	120,38	134,91	144,33	101,50	115,97	127,53	106,95	116,19	116,48
Right-subiculum-head	162,60	168,90	179,77	152,75	162,52	209,95	139,48	153,74	165,63
Right-hippocampal-fissure	129,21	156,83	176,64	129,99	145,97	177,82	157,55	174,71	181,53
Right-presubiculum-head	120,24	124,55	130,10	114,68	122,72	137,26	97,79	103,86	111,43
Right-CA1-head	468,10	497,89	515,16	408,69	489,88	550,16	404,96	406,80	451,56
Right-presubiculum-body	120,59	142,50	159,76	131,40	138,62	164,60	102,09	119,09	125,73
Right-parasubiculum	47,47	55,66	59,12	42,65	54,70	58,14	41,36	43,47	54,77
Right-molecular_layer_HP-head	296,08	317,68	330,48	260,89	313,37	345,34	245,13	270,76	284,26
Right-molecular_layer_HP-body	214,72	226,98	233,65	185,91	213,15	232,77	171,30	187,86	197,45
Right-GC-ML-DG-head	137,93	150,42	162,49	120,46	145,27	160,06	110,37	119,74	131,23
Right-CA3-body	91,21	98,27	110,08	72,78	96,03	102,93	69,77	78,87	97,74
Right-GC-ML-DG-body	128,99	138,42	141,71	113,36	133,67	142,34	101,22	113,86	126,87
Right-CA4-head	118,42	124,33	128,63	102,83	122,65	132,20	94,95	106,56	112,89
Right-CA4-body	115,61	122,75	127,75	101,87	117,86	128,39	94,62	103,76	119,52
Right-fimbria	49,05	64,06	68,75	57,14	65,34	75,66	35,67	38,52	51,75
Right-CA3-head	110,40	122,40	131,03	100,42	107,85	141,23	83,16	94,62	106,72
Right-HATA	49,63	54,12	63,72	43,51	52,05	55,31	33,81	37,70	47,00
Right-Whole_hippocampal_body	1093,95	1157,32	1194,10	1055,86	1109,13	1169,87	857,33	985,31	1024,00
Right-Whole_hippocampal_head	1514,32	1600,39	1683,31	1320,59	1616,81	1726,64	1239,08	1371,61	1458,77
Right-Whole_hippocampus	3156,39	3334,20	3488,78	2814,99	3221,46	3397,52	2651,77	2877,29	2921,25

**Table 8:** Measurements of brain morphometry: Amygdala.


AMYGDALA	Control			Schizophrenics No-Dementia			Schizophrenics With-Dementia		
	Q1	Q2	Q3	Q1	Q2	Q3	Q1	Q2	Q3
Left-Lateral-nucleus	578,54	616,36	687,20	592,51	608,93	675,114	500,8847	542,393	602,047
Left-Basal-nucleus	365,58	419,43	448,13	375,68	390,22	451,7485	299,8262	337,067	435,981
Left-Accessory-Basal-nucleus	218,93	257,65	269,27	225,15	236,98	251,3289	178,3646	197,486	232,768
Left-Anterior-amygdaloid-area-AAA	48,821	51,55	56,21	45,80	50,54	55,55,387	37,17,302	41,6563	48,61
Left-Central-nucleus	37,979	45,46	51,60	40,35	40,86	46,374	33,23,594	39,811	41,8188
Left-Medial-nucleus	18,915	25,89	32,72	19,349	21,13	27,08,808	12,9617	17,6089	20,3884
Left-Cortical-nucleus	23,89	25,32	30,25	21,37	25,28	29,82,137	17,467	21,6682	24,1505
Left-Corticoamygdaloid-transitio	150,68	170,37	177,56	149,72	155,46	170,729	121,0021	127,736	154,925
Left-Paralaminar-nucleus	42,08	45,41	51,75	41,14	44,19	50,88,777	36,90,561	41,8218	52,3714
Left-Whole_amygdala	1467,48	1644,19	1764,31	1523,04	1562,22	1769,665	1203,101	1383,87	1660,44
Right-Lateral-nucleus	586,72	633,52	699,25	563,13	631,89	713,1717	553,154	572,032	591,267
Right-Basal-nucleus	373,94	425,57	469,27	359,64	399,03	464,0564	331,8074	346,88	393,728
Right-Accessory-Basal-nucleus	236,00	266,57	288,01	221,10	246,67	276,8326	178,8142	220,475	234,46
Right-Anterior-amygdaloid-area-AAA	50,19	53,568	55,93	44,02	50,89	59,61,561	43,98,011	46,4302	48,2353
Right-Central-nucleus	40,69	46,06	59,01	39,58	43,14	49,07,619	33,4057	43,4834	47,8345
Right-Medial-nucleus	22,47	28,19	35,32	16,51	22,82	28,15,544	16,84,998	25,0023	27,1092
Right-Cortical-nucleus	25,76	27,63	30,85	19,85	27,46	28,04,321	20,25,293	24,372	26,2254
Right-Corticoamygdaloid-transitio	153,79	172,16	182,31	142,31	147,39	173,973	131,9312	141,553	155,477
Right-Paralaminar-nucleus	41,17	46,95	48,91	39,92	42,19	52,60,003	39,98,902	41,0332	42,3543
Right-Whole_amygdala	1525,33	1717,07	1828,66	1440,44	1604,96	1822,632	1368,176	1452,15	1565,51

**Table 9:** Measurements of brain morphometry: White matter segmentation.


WHITE MATTER SEGMENTATION	Control			Schizophrenics No-Dementia			Schizophrenics With-Dementia		
	Q1	Q2	Q3	Q1	Q2	Q3	Q1	Q2	Q3
Left-bankssts	2559,4	2792	3157,3	2086,4	2989,35	3116	2124,5	2333,05	3004,5
Left-caudalmiddlefrontal	5815,3	6396,15	6943,7	5881,5	6276,85	7958,1	6095,3	6275,1	7311,3
Left-entorhinal	781,1	970,45	1102,7	918,1	1052,85	1114,9	695	880,8	1053,6
Left-fusiform	5763,6	6113,9	6915,3	6325,1	7323,25	7572,8	5422,5	5913,85	6705,8
Left-inferiorparietal	8849,2	10,201,4	11,177,9	9215,2	10,554,15	11,492,1	8908,1	9726,75	10,841
Left-inferiortemporal	5310,7	5790,9	6943,1	5449,9	6587,4	7385,2	5189,2	5970,2	6269,2
Left-lateraloccipital	8601,4	10,220,6	10,551,3	8787,8	11,996,75	13,442,2	9423,7	9892,3	10,681,9
Left-lateralorbitofrontal	6044,4	6308,5	6853,1	6678,4	6782,35	7637,7	5811,8	6249,2	7240,9
Left-lingual	4758,7	5519,55	6084,3	4986,1	6061,3	6295,3	5033,4	5387,7	5832,9
Left-medialorbitofrontal	3497,7	3786,05	4164,6	3735,5	4025,45	4357,5	3176,6	3439,9	3609,5
Left-middletemporal	4708,9	5261,6	5541,2	5034,3	5672,5	6766,9	4869	5024,95	5716,5
Left-parahippocampal	1314,3	1398,65	1522,8	1387,3	1562,05	1778,8	1242,1	1449,1	1515,8
Left-paracentral	3988,7	4211,35	4597,4	3916,1	4710,5	5279,9	4138,6	4201,35	4579,4
Left-parsopercularis	3066,5	3258,45	3602,9	3049,9	3615,5	4078,1	3240,5	3362,3	3587
Left-parsorbitalis	871,8	969,95	1106,2	990,7	1077,7	1160	859,1	978,05	1095,1
Left-parstriangularis	2723,2	3032,25	3387	2933,9	3214,8	3295,7	2770,8	3002,05	3374,9
Left-pericalcarine	2901,5	3245,2	4158,9	2912	3319,1	3890,9	2755,1	3134,65	3634,5
Left-postcentral	6919,6	7592,25	8023,3	6627,5	7625,15	8586,7	6875,9	7671	9462
Left-precentral	12,687,1	13,773,35	15,180,8	12,901,1	14,055,5	15,866,8	12,904,3	15,468,4	16,425,9
Left-precuneus	8340,7	8463,65	9292,4	8904,5	10,083	11,234	8547,4	9006,9	9323,9
Left-rostralmiddlefrontal	10,725,5	12,192,25	13,929,2	10,543,9	13,222,85	13,715,7	11,758,4	12,475,3	14,455,9
Left-superiorfrontal	16,004,1	17,885,95	19,425,7	18,078,9	19,415,9	21,355,1	16,288,8	17,944,95	18,573,1
Left-superiorparietal	11,526,7	12,242,5	13,505,2	10,870,2	12,901,7	14,955,8	11,336,1	11,733,75	12,688,6
Left-superiortemporal	6532,1	7137	7750,5	7601,1	8083,05	9393	5928,7	7700,6	9118,2
Left-supramarginal	7823,5	8313,95	9193,1	8054,1	9474,2	10,482,6	7807,1	8825,7	10,586,9
Left-frontalpole	252,4	273,9	288,4	235,9	261,35	322,3	221	259,1	301
Left-temporalpole	577,8	665,2	815,3	657,6	751,7	774,8	644,3	664,9	734
Left-transversetemporal	641,1	730,2	840,7	709,3	791,65	924,4	678	769,1	967,8
Left-insula	8970,9	9336,3	10,145,7	9007,7	9643,25	10,369,7	8726,8	9567,75	9766,6
Left-UnsegmentedWhiteMatter	26,706,6	28,244,75	36,486,9	25,517,6	29,610,15	34,742,9	21,842,2	23,454,2	31,774,7
Left-CerebralWhiteMatterVol	211,256,8	220,292,9	235,519,8	218,415,9	238,384,1	259,809,4	197,088,3	223,277,6	247,214,9
Right-bankssts	2302,3	2740,8	3017,3	2035,1	2117,65	2771,2	2361,4	2583,9	2862,3
Right-caudalmiddlefrontal	5527,6	5721,6	6356,6	5038,7	5838,7	7153,9	5312,6	5908,5	6403,4
Right-entorhinal	676,6	791,65	924,2	561,1	873,3	989,4	640,3	782,9	981,7
Right-fusiform	5680,9	5960,4	6509,9	6479,9	6583,95	7527,6	5623,9	6245,65	6772,5
Right-inferiorparietal	9961,5	10,736,55	12,504,4	10,619,5	11,355,65	13,636,5	9491,4	11,268	12,455,7
Right-inferiortemporal	5110,3	5547,15	6065,4	5653,5	6496,8	6902	5221,1	5896	6548,5
Right-lateraloccipital	9430,7	10,291,1	11,562,5	10,172,9	11,907	14,733,5	9391,7	10,546,7	11,832,3
Right-lateralorbitofrontal	6257,5	6956,35	7383,8	6632	7106,15	7518,7	6011,2	6464,95	7808,6
Right-lingual	4890,4	5770,35	6813,4	5691,9	5961,85	6411,9	5053,8	5664,75	6250,1
Right-medialorbitofrontal	3417	3748,75	4326,4	3766,6	3877,55	4072	3487,8	3604,05	3977,2
Right-middletemporal	5549	5746,15	5918,7	6065,7	6414,35	6945,1	5126,6	6248,65	6657,2
Right-parahippocampal	1237,7	1352,35	1508,7	1286,4	1504,2	1721,4	1255,8	1429,6	1546,4
Right-paracentral	4679,9	5016,8	6006,3	4265,3	5380,65	5970	4845,5	4901,15	5291,7
Right-parsopercularis	2853,6	3031,3	3290,4	2558,7	3080,45	3629,8	2425,9	2911,1	3032,8
Right-parsorbitalis	1056,5	1195,25	1316,7	1196,1	1361,7	1512,6	1020,1	1209,2	1313,3
Right-parstriangularis	2755,5	3245,6	3672,9	3144,7	3197,85	3831,5	2693,7	3003,55	3208,3
Right-pericalcarine	2801	3318,9	3933,8	3136,8	3491,45	3608,5	2643,8	3228,55	3490,9
Right-postcentral	6875,6	7642,45	8360,3	7082,1	7929,6	8681,3	7164,3	7847,95	8958,7
Right-precentral	13,040,3	14,191,35	16,546,8	12,177,4	14,835,85	17,251,9	14,520	15,408,75	16,389,7
Right-precuneus	8462,3	9191,15	9686,5	9097,1	10,857,25	11,248,7	9014,9	9104,25	9491,8
Right-rostralmiddlefrontal	11,747,1	12,140,8	14,056,8	12,146,9	13,261,8	14,081,9	11,655,1	12,107,85	14,164,2
Right-superiorfrontal	16,102,7	17,478,2	19,613,8	15,893,1	16,869	20,267,7	16,132,6	17,462,65	17,992,1
Right-superiorparietal	11,071,5	11,993,65	13,655,9	10,294,1	12,387,65	14,462,1	10,743,3	11,534,85	12,772,3
Right-superiortemporal	5657	6286,15	6780,7	5789,2	6919,35	7398,5	5435,2	6521,05	7169,5
Right-supramarginal	6603,8	8032,3	9347,9	8398,4	9084,95	9625,7	8161,6	8646,3	8901,4
Right-frontalpole	303,7	377,2	412,5	328,1	344,85	392,8	273,3	310,8	439,1
Right-temporalpole	637,8	644,7	711,5	624,9	763	854,5	572	737,3	901,5
Right-transversetemporal	513,7	589,3	680,8	545,7	598	673,9	582,4	604,65	726,4
Right-insula	8265	9123,95	9619,2	9641,1	10,073,15	11,061,3	8667,3	9419,45	10,227,8
Right-UnsegmentedWhiteMatter	25,960,6	27,538	35,765,9	24,765,8	29,643,45	34,291,2	23,099,9	24,085	30,091,1
Right-CerebralWhiteMatterVol	208,404,9	219,276	238,277,4	218,408,7	240,027,2	256,039,6	199,074,1	220,865,4	243,175,5

**Table 10:** Measurements of brain morphometry: Thalamus.

THALAMUS	Control			Schizophrenics No-Dementia			Schizophrenics With-Dementia		
	Q1	Q2	Q3	Q1	Q2	Q3	Q1	Q2	Q3
Left-AV	101,80	123,22	132,40	89,80	113,67	131,14	81,70	95,21	101,78
Left-CeM	54,70	58,50	60,33	51,03	54,05	59,68	39,31	44,02	50,16
Left-CL	27,60	32,81	41,01	25,00	30,63	41,87	25,17	27,25	31,99
Left-CM	213,50	224,75	245,7	210,35	256,31	270,21	213,80	223,26	250,52
Left-LD	20,59	25,714	35,71	14,31	25,69	27,33	11,53	16,547	22,48
Left-LGN	134,75	155,17	175,9	130,86	142,47	153,46	102,18	114,07	124,41
Left-LP	104,07	113,62	131,6	91,27	110,52	116,20	91,01	99,43	111,24
Left-l-Sg	15,95	17,44	22,6	20,36	22,16	29,27	21,45	24,09	31,41
Left-MDl	238,92	252,15	268,30	225,12	241,727	248,55	187,25	193,19	225,14
Left-MDm	658,13	685,42	734,30	586,02	613,68	671,23	462,79	511,97	537,73
Left-MGN	105,67	110,73	115,20	81,98	96,83	113,62	86,70	92,98	95,47
Left-MV(*Re*)	10,10	11,197	11,95	8,56	9,73	11,80	6,73	8,05	8,90
Left-Pc	2,92	3213	3447	2,89	3,06	3,22	2,17	2,77	3,13
Left-Pf	44,56	50,183	55,92	51,08	58,46	61,39	44,85	50,36	53,87
Left-Pt	6,10	6,53	7,04	5,94	6629	7,01	5,84	6,23	6,75
Left-PuA	174,10	182,46	188,90	157,83	175,55	186,80	137,81	150,07	162,49
Left-PuI	142,61	151,28	164,10	133,57	154,53	173,27	123,37	138,72	144,1
Left-PuL	120,19	123,13	134,60	116,87	134,74	138,25	110,79	125,09	131,06
Left-PuM	805,67	854,67	954,00	750,39	779,00	909,25	697,39	736,55	810,46
Left-VA	326,46	364,82	401,80	361,29	397,81	435,44	297,04	340,52	398,93
Left-VAmc	26,87	28,55	32,51	28,53	31,07	35,41	25,08	27,04	30,246
Left-VLa	508,02	559,38	628,90	576,84	585,97	687,71	475,83	534,23	581,85
Left-VLp	671,06	699,26	798,30	706,29	759,24	896,26	619,69	672,08	776,72
Left-VM	17,06	18,56	19,88	17,76	19,64	22,12	16,74	17,59	18,63
Left-VPL	767,91	783,85	816,10	721,47	844,39	930,17	684,95	734,57	813,46
Left-Whole_thalamus	5452,16	5652,60	6021,00	5123,95	5622,44	6492,57	4699,14	4979,50	5216,80
Right-AV	114,56	130,84	141,20	103,37	107,52	126,78	83,87	104,28	119,66
Right-CeM	55,1786	62,124	65,77	49,71	55,45	66,29	39,66	48,06	54,14
Right-CL	28,4528	33,636	38,68	25,72	28,65	30,98	20,51	26,14	29,84
Right-CM	207,12	213,33	224,7	211,55	221,67	249,89	200,80	208,55	229,20
Right-LD	20,61	25,835	31,53	13,32	18,26	23,46	9,96	12,13	17,65
Right-LGN	158,94	183,38	195,3	161,84	164,85	191,54	126,30	134,90	156,92
Right-LP	102,14	105,65	109,5	87,85	95,88	108,26	70,78	83,27	98,89
Right-l-Sg	13,75	16,47	17,53	16,16	18,80	22,73	13,49	18,71	24,69
Right-MDl	251,41	269,95	280,90	233,42	253,13	272,55	211,71	230,65	247,19
Right-MDm	668,64	708,86	745,50	569,77	633,18	682,75	516,25	593,92	604,94
Right-MGN	105,49	114,92	128,20	97,05	117,11	126,36	88,91	102,66	110,76
Right-MV(*Re*)	9,35	11,02	12,21	8,41	9,39	12,43	5,89	6,72	7,37
Right-Pc	2,76	3,37	3,61	2,73	2,94	3,28	2,38	2,59	2,81
Right-Pf	40,71	48,73	53,51	47,23	54,78	57,38	44,85	47,45	49,90
Right-Pt	5,50	5,9962	6,48	5,48	6,09	6,80	5,02	5,43	6,34
Right-PuA	198,37	208,89	225,50	181,20	223,54	238,50	177,43	193,52	203,60
Right-PuI	163,84	182,63	199,00	163,62	203,49	217,09	162,94	177,77	202,67
Right-PuL	145,41	161,89	178,70	157,75	188,44	234,294	156,39	163,00	204,13
Right-PuM	913,46	956,59	1005,00	825,22	1002,70	1035,26	784,75	898,11	1005,40
Right-VA	317,86	340,85	409,80	333,31	370,32	407,49	311,06	339,95	356,22
Right-VAmc	26,73	29,644	33,08	27,84	30,86	34,0172	24,26	28,17	29,98
Right-VLa	479,02	541,25	640,90	550,45	579,21	648,80	477,29	519,28	552,66
Right-VLp	630,31	700,26	806,30	703,53	737,55	838,49	631,17	657,73	697,03
Right-VM	16,58	17,457	20,68	17,60	19,59	23,54	15,19	17,50	17,973
Right-VPL	719,83	742,18	807,80	726,38	810,50	929,17	650,30	704,24	800,15
Right-Whole_thalamus	5487,96	5814,00	6284,00	5375,49	5865,79	6453,36	4944,68	5178,10	5721,30

**Table 11:** Measurements of brain morphometry: Choroid plexus.

CHOROID PLEXUS	Control			Schizophrenics No-Dementia			Schizophrenics With-Dementia		
	Q1	Q2	Q3	Q1	Q2	Q3	Q1	Q2	Q3
lf-vessel	12,30	34,20	58,20	19,80	25,70	32,40	11,80	23,10	30,20
lf-choroid-plexus	446,70	498,00	539,20	633,00	845,10	999,80	735,50	808,30	839,80
rf-vessel	13,70	30,00	34,40	13,60	20,40	34,20	16,50	23,750	37,40
rf-choroid-plexus	414,60	458,20	702,70	520,30	714,85	927,10	632,50	689,05	774,10

**Table 12:** Measurements of brain morphometry: Gray matter segmentation.


GRAY MATTER SEGMENTATION	Control			Schizophrenics No-Dementia			Schizophrenics With-Dementia		
	Q1	Q2	Q3	Q1	Q2	Q3	Q1	Q2	Q3
Left-Unknown	4612,30	4989,70	5478,70	5025,00	5521,85	5756,60	4996,20	5193,00	5507,90
Left-Bankssts	782,90	905,50	1029,90	829,00	984,85	1137,90	760,70	890,65	949,50
Left-Caudalanteriorcingulate	481,90	594,10	728,50	478,80	505,65	590,50	506,20	530,20	609,00
Left-Caudalmiddlefrontal	1891,50	2059,10	2227,50	1884,50	2044,2	2502,10	1943,60	2001,25	2237,100
Left-Cuneus	1213,00	1363,00	1483,00	1304,70	1498,85	1855,60	1241,20	1305,20	1533,10
Left-entorhinal	382,40	449,90	525,30	416,30	520,45	562,50	415,30	432,65	497,30
Left-Fusiform	2654,10	2910,00	3437,60	2745,20	3071,90	3461,90	2527,50	2601,20	2874,30
Left-inferiorparietal	3693,00	4332,60	4870,50	3863,3	4299,70	4476,10	3658,40	4134,10	4613,40
Left-Inferiortemporal	2508,40	3149,70	3718,50	3206,90	3341,75	3586,40	2563,60	2881,70	3408,90
Left-isthmuscingulate	802,9 0	975,70	1132,20	866,30	963,30	1136,70	923,50	1024,05	1138,60
Left-lateraloccipital	4182,10	4822,90	5056,20	4025,70	5279,15	6054,90	4228,70	4579,85	4965,00
Left-lateralorbitofrontal	2206,80	2460,20	2638,70	2431,50	2522,65	2822,70	2300,40	2368,55	2558,10
Left-Lingual	2608,00	2791,20	3134,60	2386,70	2587,20	3009,8	2342,70	2596,80	3233,20
Left-medialorbitofrontal	1753,00	1866,2	2012,9	1804,2	1975,4	2171,3	1719,6	1771	1917,6
Left-middletemporal	2495,40	2847,4	3129,1	2691	3034,35	3303,2	2480,5	2749,9	3116,2
Left-parahippocampal	565,60	584	601,6	618,1	678,65	769,7	537,7	602,45	613
Left-paracentral	1260,20	1386,6	1477,4	1212,3	1399,5	1429,9	1243,5	1329,5	1514,6
Left-parsopercularis	1314,90	1538	1658,5	1287,9	1610,35	1788,2	1390,1	1418,9	1533
Left-parsorbitalis	555,50	631,7	700,5	629,2	651,45	687,1	551,7	613,85	645,5
Left-parstriangularis	1051,5	1279,8	1435,6	1095,1	1258,55	1312,4	1113,4	1176	1277,3
Left-pericalcarine	1175,4	1255,6	1505,4	1080,8	1330,025	1416,7	977,7	1208,8	1396,8
Left-postcentral	3579,4	3908,7	4156,7	3459,4	3723,25	4338,9	3617	3803,25	4097,1
Left-posteriorcingulate	973,7	1133	1279,6	974,6	1039,8	1114,8	950,7	1017,85	1122,6
Left-precentral	4234,1	4634,8	5029	4325,2	4701,05	5381,4	4313,7	4719,85	5102,4
Left-precuneus	3099,7	3379,6	3702,4	3638,1	3899,6	4003,2	3273,6	3383,05	3616,2
Left-rostralanteriorcingulate	719,9	764,3	886,5	684	811,65	991,4	659,3	750,2	774,4
Left-rostralmiddlefrontal	4621,7	5189,6	5736,5	4309,2	5300,6	5751,1	4472,4	5078,2	5516,1
Left-superiorfrontal	6122,1	6869,1	7790,5	5955,3	7047,1	7256,2	6237,4	6626,4	7205,7
Left-superiorparietal	4855,5	5163,9	5647,2	4860,8	5594,1	6186,5	4510,9	4776,15	5116,2
Left-superiortemporal	3358,1	3671,7	3836,5	3726,5	4052,75	4199,7	3235,5	3430,35	3983,9
Left-supramarginal	3308,6	3450,1	3915	3464,6	4094,706	4401,8	3320,1	3838,75	4225,4
Left-frontalpole	207,9	227,9	249,3	205,3	237,4	250,2	194,7	228,5	241
Left-temporalpole	397,3	466,9	505,4	464,1	500,15	520,9	437,5	453,65	496,7
Left-transversetemporal	359,2	389,1	415,2	381	464,55	503,8	351,9	358,6	452,1
Left-Insula	2033,1	2215,2	2366,6	2115,6	2272,25	2574,8	2191,1	2310,5	2528,2
Right-Unknown	4754,1	4884,25	5350,1	5044,1	5326,9	5543,2	5067,8	5285,9	5552
Right-Bankssts	738,1	801,95	889,3	670,5	781,55	866,6	765	837,9	918,6
Right-caudalanteriorcingulate	515,2	651,65	837	679,9	753,15	844	519,4	609,5	682,7
Right-caudalmiddlefrontal	1774,2	1958,8	2237,1	1706,4	1815,85	2277,9	1716,2	2000,4	2428,9
Right-Cuneus	1248,9	1491,4	1783,2	1464,6	1713	1752,8	1316,8	1488,6	1726,2
Right-entorhinal	344,4	384	432,7	304,9	475,8	510,1	334,5	390,9	461,3
Right-Fusiform	2449,7	2777,35	3126,3	2692,6	2973,9	3071,9	2353,9	2706,3	2880,1
Right-inferiorparietal	4233,7	4952,9	5694,2	4379	4908	5586,1	4431,7	4514,7	5540
Right-inferiortemporal	2759,5	3010,7	3414,2	3027,4	3340,45	3515,9	2704,9	3033,6	3277,8
Right-isthmuscingulate	772,9	878,1	957,8	778,8	933,7	997,7	791,8	874,6	951,9
Right-lateraloccipital	4333,1	4668,55	5351,1	4085,5	5143,3	6300,1	4237,1	4595,1	5030,3
Right-lateralorbitofrontal	2275,6	2499,4	2705,9	2371,1	2612,95	2835,2	2306,9	2439,4	2529,2
Right-Lingual	2582,4	3037,45	3317	2650,2	2870,65	3091,3	2520,5	2882,8	3149,2
Right-medialorbitofrontal	1674,6	1821,25	2190,8	1803,6	1880,85	2079,2	1680,3	1753	1937,4
Right-middletemporal	2885,3	3171	3484,5	3010,5	3236,5	3525,2	2947,7	3189,7	3556,1

**Table:** **Table 12** (*continued*)


GRAY MATTER SEGMENTATION	Control			Schizophrenics No-Dementia			Schizophrenics With-Dementia		
	Q1	Q2	Q3	Q1	Q2	Q3	Q1	Q2	Q3
Right-parahippocampal	509,1	558,75	607,4	561,1	650,1	712,8	513,9	556,8	610,1
Right-paracentral	1377,2	1514,9	1732,2	1372,5	1495,35	1605,8	1420,9	1497,9	1548,5
Right-parsopercularis	1217,3	1321,2	1409,7	1161,1	1293,25	1433,3	1040,4	1146,9	1268,7
Right-parsorbitalis	658,8	683,95	793,3	729,2	836,15	925,6	681,4	725,1	754,1
Right-parstriangularis	1236,1	1383,45	1653,6	1216,6	1431,75	1621	1156,8	1277,4	1391,5
Right-pericalcarine	1234,3	1367,4	1493,2	1271,2	1376,45	1516,1	1112,3	1370,6	1409,3
Right-postcentral	3476	3662,45	4124,1	3628,7	3716,65	4032,7	3680,3	3779,5	4102,6
Right-posteriorcingulate	1063,5	1128,4	1261,2	982,5	1118,55	1215,1	964,2	1030,3	1159,1
Right-precentral	4217,3	4614,85	5394,7	4062,6	4904,9	5260,7	4414,4	4767,8	5345
Right-precuneus	3342,2	3559,45	3767,4	3475,2	4023,45	4126,5	3331,5	3575,9	3706,4
Right-rostralanteriorcingulate	479	547,65	658,5	555,9	622,05	750,6	454,9	570,3	601,1
Right-rostralmiddlefrontal	4921,4	5104,7	5753,1	4589,6	5338,2	5682,1	4639,5	5151,7	5841,4
Right-superiorfrontal	6150,2	6472,2	7739,7	5554,4	6027,1	6748,6	5969,3	6421,8	6841,2
Right-superiorparietal	4399,5	5155,05	5599	4618,5	5339,75	5611,5	4493,6	4827,6	5372,1
Right-superiortemporal	3164,2	3359,45	3719,7	3195,2	3640,95	3927,3	3180,7	3416,8	3958,1
Right-supramarginal	2714,5	3123,7	3818,5	3447,4	3748,9	4023	3258,4	3361	3770,4
Right-frontalpole	258,9	294,5	333,1	274,9	289,6	314	233,7	266	295,7
Right-temporalpole	428,3	445,4	460,9	432,6	480,6	533,6	347,8	448,3	542,6
Right-transversetemporal	258,1	287,15	319	252,9	300,1	343,5	272,4	292,9	335,5
Right-Insula	1910,8	2115,3	2250,5	2362,5	2428,95	2566,2	2101,2	2305,6	2464

## 2 Experimental Design, Materials, and Methods

An observational, descriptive-comparative study was conducted from an exploratory perspective. Considering the characteristics of the study, only ambulatory patients were included.

### 2.1 Sampling design

□ Population: Patients schizophrenic without dementia and schizophrenic with dementia from Hospital Psiquiátrico Universitario del Valle and control patients from the Fundación Valle del Lili.
□ Sampling strategy: Stratified non-random, three strata were considered: control group, schizophrenia without dementia and schizophrenia with dementia.
□ Sample size: 35 patients were distributed into: 15 control group, 10 schizophrencs without dementia and 10 schizophrenics with dementia.
□ Sample sex distribution: control group {9 Females, 6 Males}, schizophrenics without dementia group {5 Females, 5 Males}, schizophrenics with dementia group {4 Females, 6 Males}.
□ Sample features: The control group was taken from the Mild Cognitive Impairment progression study. The onset of symptoms was before age 30 in all patients. Patients in the schizophrenics without dementia group were included who met the criteria of the Diagnostic and Statistical Manual of Mental Disorders in the fifth edition (DSM-V) for schizophrenia, had a mini-mental (MMSE) greater than or equal to 26 and a Clinical Dementia Rating (CDR) score of less than 0,5 (Non-Dementia), Patients in the schizophrenics with dementia group met the DSM-V criteria for schizophrenia and had an MMSE less than 26 and a CDR greater than 1. The control group had no history of mental illness and the MMSE was greater than 26 and the CDR was 0.
□ Age: All patients over 45 years old.

### 2.2 Neuropsychological tests

Two general screening tests were used for neuropsychological assessment, the Mini-Mental State Examination (MMSE) (Folstein 1975) and the Addenbrooke's Cognitive Examination (ACE) (Bak 2007). These tests allowed an overall assessment of the patients' cognitive performance, as well as to locate the degree of functionality in relation to the sample. Additionally, to confirm or rule out the presence of a dementia state, the Clinical Dementia Rating (CDR) Scale (Morris 1993) and the Hachinski Ischemic Score (Hachinski 1975) were performed. All participants also answered the Yesavage Geriatric Depression Scale (Yesavage 1983), with the aim of analysing possible concomitance. In schizophrenic patients the Positive and Negative Symptom Scale in Schizophrenia (PANSS) was applied (Kay 1987).

The Hopkins Verbal Learning Test (Brandt 1991), the Rey Complex Figure test (Osterrieth 1944), and the Free and Cued Selective Reminding Test (FCSRT) (Buschke 1974) were used to analyze the mnemic component. The Hopkins Verbal Learning and the FCSRT assess learning and memory capacity at the audio-verbal level, while the Rey Complex Figure test assesses the coding and evocation of visual graphic material. It is important to consider that both tests quantify short-term memory and are not sensitive for measuring the ability to consolidate information in the long term. The Boston Naming Test (Kaplan 1983) was used to test language function. In addition to directly examining the subject's nominative capacity, this test implicitly indicates the degree of deterioration or preservation of semantic memory.

In relation to the functions associated predominantly with the prefrontal cortex, the Trail Making Test - A (TMT - A) (Army Individual Test Battery 1944), the phonological fluency test (Letter F and S), the semantic fluency test (animal fluency) and the Digit Spam test were applied. The TMT-A directly assesses visual attention, and indirectly evaluates the ability to sequence, plan and structure short-term goals. The Phonological Fluency Test (Letter F and S) (Belleville 2017) and the Semantic Fluency Test (animal fluency) assess mental flexibility and the ability to categorize, however, it is also a tool for the analysis of verbal function and semantic memory. Finally, the Digit Spam test is a task involved in the assessment of working memory.

The assessments were carried out by Dr. Carlos Gonzalez and his students at the Hospital Psiquiatrico Universitario del Valle in Cali, Colombia.

### 2.3 Brain morphometry

Images acquisition: MRI scans were taking at the Image Diagnostic Unit of the Fundación Valle del Lili, in July 2017, using a SIEMENS Avanto 1,5 T with the following characteristics: Series description: t2_flair_blade_tra, Series number: 4. Repetition time (ms): 8000. Echo time[0] (ms). Echo time[1] (ms): 99. Inversion time (ms): 2371,2. Flip angle: 150. Number of averages: 1. Slice thickness (mm): 5. Slice spacing (mm): 6. Image columns: 256. Image rows: 256. Phase encoding direction: ROW. Voxel size x (mm): 0,898438. Voxel size y (mm): 0,898438. Orientation: tra.

Images processing: Cortical reconstruction and volumetric segmentation was performed with the FreeSurfer version V6 image analysis suite, which is available online at http://surfer.nmr.mgh.harvard.edu/ [5]. FreeSurfer's cortical segmentation provides measures of surface area, cortical thickness, gray matter volume, white matter volume, and cortical mean curvature. Volumes, in mm^3^, were obtained using FreeSurfer's subcortical segmentation.

Using longitudinal recon-all, images were processed with the longitudinal stream [8] to extract volume estimates. Initially a registration is done [7]. After that, several processing steps, such as skull stripping, Talairach transforms, atlas registration as well as spherical surface maps and parcellations are done [8]. After running recon-all, the separate hippocampus/amygdala pipelines was used for segmentation of the subcortical white matter and deep gray matter volumetric structures (including hippocampus, amygdala) [1,3,4,9]. Finally, Thalamus segmentation was done after running recon-all [2].

The FreeSurfer uses the following abbreviations:

**Table 13:** FreeSurfer's abbreviations for thalamus.

Abbreviation	Explanation	Abbreviation	Explanation
AV	Anteroventral nucleus	Pc	Paracentral ncl,
CeM	Central medial nucleus	Pf	Parafascicular ncl,
CL	Central lateral nucleus	PuA	Ncl. pulvinaris anterior
CM	Centromedian nucleus	Pul	Ncl. pulvinaris inferior
LD	Lateral dorsal nucleus	PuL	Ncl. pulvinaris lateralis
LGN	Lateral geniculate nucleus	PuM	Ncl. pulvinaris medialis
LP	Lateral posterior nucleus	VA	Ncl. ventralis anterior
L-Sg	Limitans/suprageniculate ncl	VamC	Ncl. ventralis anterior, pars magnocellularis
MDI	Multiform (lateral) division of MD	Vla	Ventral lateral anterior nucleus
Mdm	Mediodorsal ncl. magnocellular division	Vlp	Ventral lateral posterior nucleus
MGN	Medial geniculate nucleus	VM	Ventral medial nucleus
MV(*Re*)	Medioventral ncl. (reuniens ncl.)	VPL	Ventral posterior lateral nucleus

## Declaration of Competing Interest

The authors declare that they have no known competing financial interests or personal relations that could have appeared to influence the work reported in this paper.

## Appendix A: Supplementary data

Supplementary data to this article can be found online at https://zenodo.org/record/3901876#.Xu1w2G5FxPb.

## Appendix B: Subject selection criteria

### Inclusion criteria

Schizophrenic subjects with dementia:

• Men and women over 45 years of age.
• Mini mental state examination (MMSE) less than 26
• Clinical diagnosis of schizophrenia before the age of 30
• Meet DSM V criteria for major neurocognitive disorder:
  ○ Evidence of significant cognitive decline compared to previous level of performance in one or more cognitive domains (complex attention, executive function, learning and memory, language, motor perceptual ability, or social cognition) based on:
    ■ Concern in the individual himself, in an informant who knows him or in the clinician, because there has been a significant decline in a cognitive function and
    ■ A substantial decline in cognitive performance, preferably documented by a standardized neuropsychological test or, failing that, by another quantitative clinical assessment.
  ○ Cognitive deficits interfere with an individual's autonomy in daily activities (i.e., he or she needs assistance with complex instrumental activities of daily living, such as paying bills or complying with treatments).
  ○ Cognitive deficits do not occur exclusively in the context of a confusional syndrome.
  ○ Cognitive deficits are not better explained by another mental disorder (e.g., major depressive disorder, schizophrenia).
• Score greater than 1 on the Clinical Dementia Rating Scale
• Sign the informed consent

### Schizophrenic subjects without dementia

• Women and men over the age of 45.
• Mini mental state examination (MMSE) greater than or equal to 26
• Clinical diagnosis of schizophrenia before the age of 30
• Meet DSM V criteria for schizophrenia:
  a Two (or more) of the following symptoms, each present for a significant part of the time over a period of one month (or less if successfully treated). At least one of them must be (1), (2), or (3): 1. 2. hallucinations 3. disorganized speech (e.g., frequent disintegration or incoherence) 4. highly disorganized or catatonic behavior 5. negative symptoms (i.e., diminished emotional expression or abulia)
  b For a significant portion of time since the onset of the disorder, the level of functioning in one or more major domains, such as work, interpersonal relationships, or personal care, is well below the level achieved before the onset (or, when it begins in childhood or adolescence, fails to achieve the expected level of interpersonal, academic, or work functioning).
  c Continuous signs of the disorder persist for at least six months. This six-month period must include at least one month of symptoms (or less if successfully treated) that meet the first criterion (a.) (i.e., active phase symptoms) and may include periods of prodromal or residual symptoms. During these prodromal or residual periods, the signs of the disorder may be manifested by negative symptoms only or by two or more symptoms listed in Criterion A being present in an attenuated form (e.g., bizarre beliefs, unusual perceptual experiences).
  d Schizoaffective disorder and depressive or bipolar disorder with psychotic features have been ruled out because
    i no major manic or depressive episodes have occurred concurrently with the active-phase symptoms, or
    ii if mood episodes have occurred during the active-phase symptoms, they have been present for only a minimal portion of the total duration of the active and residual periods of illness.
  e The disorder cannot be attributed to the physiological effects of a substance (e.g., a drug or medication) or to another medical condition.
  f If there is a history of an autism spectrum disorder of a childhood-onset communication disorder, further diagnosis of schizophrenia is only made if the noticeable delusions or hallucinations, in addition to the other symptoms required for schizophrenia, are also present for at least one month (or less if successfully treated).
• Not meeting DSM V criteria for major neurocognitive disorder:
  a Evidence of significant cognitive decline compared to previous level of performance in one or more cognitive domains (complex attention, executive function, learning and memory, language, motor perceptual ability, or social cognition) based on:
    i Concern in the individual himself, in an informant who knows him or in the clinician, because there has been a significant decline in a cognitive function and
    ii A substantial decline in cognitive performance, preferably documented by a standardized neuropsychological test or, failing that, by another quantitative clinical assessment.
  b Cognitive deficits interfere with an individual's autonomy in daily activities (i.e., he or she needs assistance with complex instrumental activities of daily living, such as paying bills or complying with treatments).
  c Cognitive deficits do not occur exclusively in the context of a confusional syndrome.
  d Cognitive deficits are not better explained by another mental disorder (e.g., major depressive disorder, schizophrenia).
• Score of 0.5 or 0 on the Clinical Dementia Rating Scale
• To coincide with the other two groups in terms of socio-demographic variables: schooling, laterality, ethnicity, place of residence, etc.
• Sign the informed consent.

Control subjects:

• Women and men over 45 years of age.
• Mini mental state examination (MMSE) greater than or equal to 26
• No clinical diagnosis of schizophrenia.
• Not meeting DSM V criteria for major neurocognitive disorder:
  a Evidence of significant cognitive decline compared to previous level of performance in one or more cognitive domains (complex attention, executive function, learning and memory, language, motor perceptual ability, or social cognition) based on:
    i Concern in the individual himself/herself, in an informant who knows him/her or in the clinician, because there has been a significant decline in a cognitive function and
    ii A substantial decline in cognitive performance, preferably documented by a standardized neuropsychological test or, failing that, by another quantitative clinical assessment.
  b Cognitive deficits interfere with an individual's autonomy in daily activities (i.e., he or she needs assistance with complex instrumental activities of daily living, such as paying bills or complying with treatments).
  c Cognitive deficits do not occur exclusively in the context of a confusional syndrome.
  d Cognitive deficits are not better explained by another mental disorder (e.g., major depressive disorder, schizophrenia).
• Score of 0 on the Clinical Dementia Rating Scale
• To coincide with the other two groups in terms of socio-demographic variables: schooling, laterality, ethnicity, place of residence, etc.
• Sign the informed consent.

### Exclusion criteria

Schizophrenic subjects without and with dementia:

• History of neurological diseases such as epilepsy, stroke, and other neurodegenerative disorders
• History of traumatic brain injury
• History of HIV positive or neurosyphilis
• Subjects who are claustrophobic, have anxiety problems, or any other condition that prevents collaboration during the MRI
• Subjects with orthodontic material, aneurysm clips, or any metal implant
• Subjects with recent tattoos on their face or neck as some inks used have characteristics that alter the intensity of the signal
• Recent metal prostheses, or pacemakers

Control subjects:

• History of mental illness with psychotic symptoms
• History of dementia
• History of neurological diseases such as epilepsy, stroke, and other neurodegenerative disorders
• History of traumatic brain injury
• History of HIV positive or neurosyphilis
• Subjects who are claustrophobic, have anxiety problems, or any other condition that prevents collaboration during the MRI
• Subjects with orthodontic material, aneurysm clips, or any metal implant
• Subjects with recent tattoos on their face or neck
• Recent metal prostheses, or pacemakers

## Appendix C Supplementary materials

Image, application 1
